# Pros and cons of the gasless laparoscopic transhiatal esophagectomy for upper esophageal carcinoma

**DOI:** 10.1007/s00464-015-4488-z

**Published:** 2015-09-28

**Authors:** Lei Yu, Ji-xiang Wu, Yu-shun Gao, Jian-ye Li, Yun-feng Zhang, Ji Ke

**Affiliations:** Department of Thoracic Surgery, Beijing Tongren Hospital, Capital Medical University, Beijing, China; Department of Surgery, Beijing Tongren Hospital, Capital Medical University, No. 1 Dongjiaominxiang Street, Dongcheng District, Beijing, 100730 China; Department of Thoracic Surgery, Cancer Institute and Hospital, Chinese Academy of Medical Sciences, Peking Union Medical College, No. 17 Panjiayuan, Chaoyang District, Beijing, China

**Keywords:** Minimally invasive esophagectomies, Upper esophageal carcinoma, Outcome, Laryngo-pharyngeal reflux

## Abstract

**Background:**

Controversies on how to treat upper esophageal carcinoma have existed for several decades. With the application of minimally invasive techniques, surgical treatment to upper esophageal carcinoma tends to show more advantages and attract more patients. Up to now, most hospitals adopted the combined thoracoscopic and laparoscopic esophagectomy (CTLE) as the way of minimally invasive surgery for upper esophageal carcinoma. But CTLE to treat upper esophageal carcinoma has its drawbacks, such as demanding certain pulmonary function and severe postoperative regurgitation. In 2011, we developed the gasless laparoscopic transhiatal esophagectomy (LTE) to treat upper esophageal carcinoma, which showed some advantages. The aim of this article was to compare LTE with CTLE in treating upper thoracic or cervical esophageal carcinoma and assess the value of LTE.

**Methods:**

From 2009 to 2014, esophagectomy has been performed by the introduction of minimally invasive surgery in a total of 83 patients with upper thoracic or cervical esophageal carcinoma. Among these patients, LTE was performed in 27 cases (Group 1), while CTLE was performed in the other 56 (Group 2). Neoadjuvant chemotherapy was done in patients of Group 1.

**Results:**

There were no operation-related deaths and conversion to open procedure. There was no significant difference in postoperative complications, ventilation time, ICU stay, hospital stay, and anastomotic leak rates between the two groups. But LTE was associated with shorter operative time and less intraoperative blood loss. In Group 2, 21 (37.5 %) patients had postoperative pulmonary complications, while in Group 1, there were 6 (22.2 %) patients having pulmonary complications at least one time. Results of 24-h pH monitoring and manometry showed that postoperative laryngo-pharyngeal reflux (PLPR) was more severe in Group 2 patients than in Group 1; for Group 1, PLPR mainly occurred on sleep stage, while for Group 2, PLPR might exist all the day with short intervals and last longer at night. The median overall survival was 27.2 months after CTLE and 30.8 months after LTE (*P* = 0.962). There was no significant difference in survival at 2, 3 and 4 years between the two groups.

**Conclusions:**

Compared with CTLE, LTE is a more minimally invasive approach to effectively treat patients with upper esophageal carcinoma. Laryngo-pharyngeal reflux after LTE was less severe than that after CTLE, which might lower incidence of pulmonary complications. For the elderly patients, LTE seems more suitable.

Controversies on how to treat upper esophageal carcinoma have existed for several decades. Some experts believed that surgery and radiotherapy to upper esophageal carcinoma could lead to equivalent results, and both of them had pros and cons [1–7]. Radiotherapy had lower rates of morbidity and mortality [1–4], while surgery might result in long-term outcome [5–7]. But with the application of minimally invasive techniques, surgical treatment to upper esophageal carcinoma tends to show more advantages and attract more patients [8, 9]. Up to now, most hospitals adopted the combined thoracoscopic and laparoscopic esophagectomy (CTLE) as the way of minimally invasive surgery for upper esophageal carcinoma. But CTLE to treat upper esophageal carcinoma has its drawbacks, such as demanding certain pulmonary function and severe postoperative regurgitation [8, 10–12]. In 2011, the gasless laparoscopic transhiatal esophagectomy (LTE) to treat upper esophageal carcinoma was developed in our department, which showed some advantages. It could be performed for the elderly or patients with severe preoperative pulmonary dysfunction. The aim of this article was to compare LTE with CTLE in treating upper thoracic or cervical esophageal carcinoma and assess the value of LTE.

## Materials and methods

**Fig. 1:**
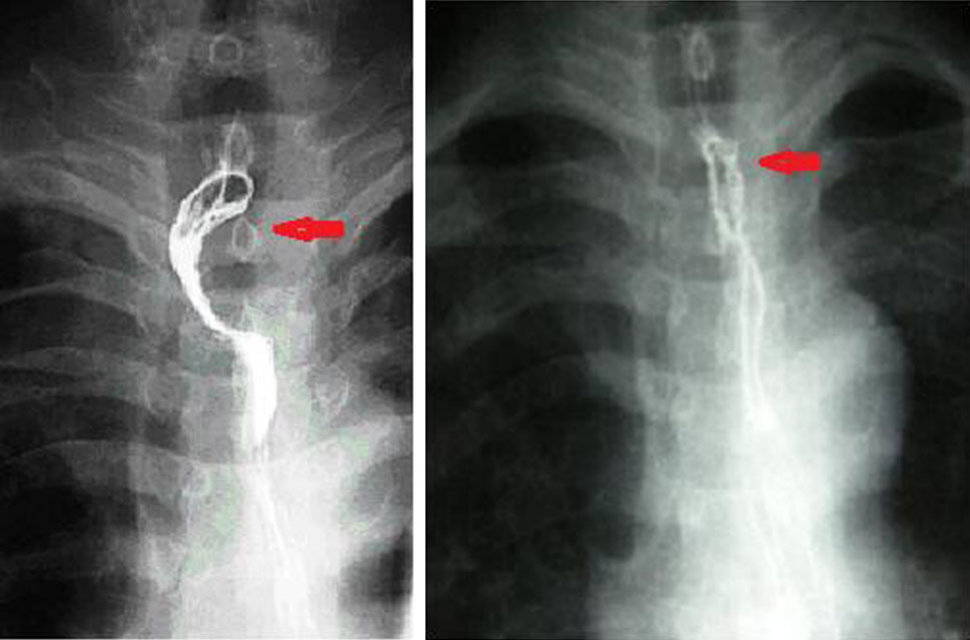
Upper esophageal carcinoma with local advance downstaged effectively after neoadjuvant chemotherapy (the right picture was taken before neoadjuvant chemotherapy; the left one after neoadjuvant chemotherapy)

From 2009 to 2014, esophagectomy has been performed by the introduction of minimally invasive surgery in a total of 83 patients with upper thoracic or cervical esophageal carcinoma. Cervical esophageal carcinoma accounted for 61.4 %, and upper thoracic esophageal carcinoma accounted for 38.6 %. Among these patients, the gasless laparoscopic transhiatal technique (LTE) was performed in 27 cases (Group 1), while a combined laparoscopic and thoracoscopic technique (CTLE) was used in the other 56 (Group 2) (Table 1). Neoadjuvant chemotherapy was done in patients of Group 1. There were 13 cases in whom observed tumors obviously shrunk after neoadjuvant chemotherapy (Fig. 1). Among them, esophageal carcinoma with local advance downstaged effectively in 11 cases. LTE was done within 4 weeks after completion of neoadjuvant chemotherapy. No one received preoperative radiotherapy.

**Table 1:** Characteristics of patients with upper esophageal cancer undergoing LTE or CTLE

	Group 1 (27 cases)	Group 2 (56 cases)
Sex		
Male	21	43
Female	6	13
Median age (range)	72 (47–89)	61 (41–78)
Tumor site		
Cervical esophagus	19	31
Upper thoracic esophagus.	8	25
Stage		
I	6	9
II	9	32
III	12	15
Mean operative time (minute)	131 ± 29	175 ± 15
Intraoperative blood loss (ml)	189 ± 52	336 ± 87
Histologic type	Squamous cell carcinoma	Squamous cell carcinoma
No. of lymph nodes dissected	7 (3–18)	18 (11–26)
Ventilation time—days (range)	1 (0–5)	2 (0–6)
ICU stay—days (range)	1 (0–6)	2 (0–7)
Hospital stay—days (range)	12 (11–27)	13 (11–33)

Endoscopy with biopsy was performed before surgery and histologic examination confirmed that all were squamous cell carcinoma. Computed tomography (CT scan), ultrasonography, barium esophagram and bronchoscopy were routinely undertaken on all patients to stage preoperatively and confirm the absence of contra-indications for thoracoscopy and laparoscopy. Endoscopic ultrasound (EUS) was done to obtain accurate locoregional staging in 56.6 % patients, and positron emission tomography scan (PET) data were obtained in 34.9 % patients. But 7 patients could not afford the costs of EUS or PET, so we had to rely on CT to judge the tumor stage.

The study was approved by the Human Research Ethics Board of Beijing Tongren Hospital, Capital Medical University, and Cancer Institute and Hospital, Chinese Academy of Medical Sciences. Patients were warned in regard to the potential risks prior to surgery and signed consent forms if they agreed to undergo esophagectomy by minimally invasive techniques.

### Follow-up

Patients with upper thoracic or cervical esophageal carcinoma received adjuvant chemotherapy after LTE or CTLE. All patients were followed up at the outpatient clinic for the first 3 months after surgery, and then in intervals of 6 months during the postoperative 5 years. In order to objectively measure postoperative reflux, postoperative 24-h pH monitoring and manometry were undertaken and documented between 6 months and 1 year after surgery.

### Surgical technique

1. The combined laparoscopic and thoracoscopic technique was performed as Luketich prescribed previously [8].
2. The gasless laparoscopic transhiatal esophagectomy was performed with five upper abdominal laparoscopic incisions and a 4-cm-long left cervical incision.

**Fig. 2:**
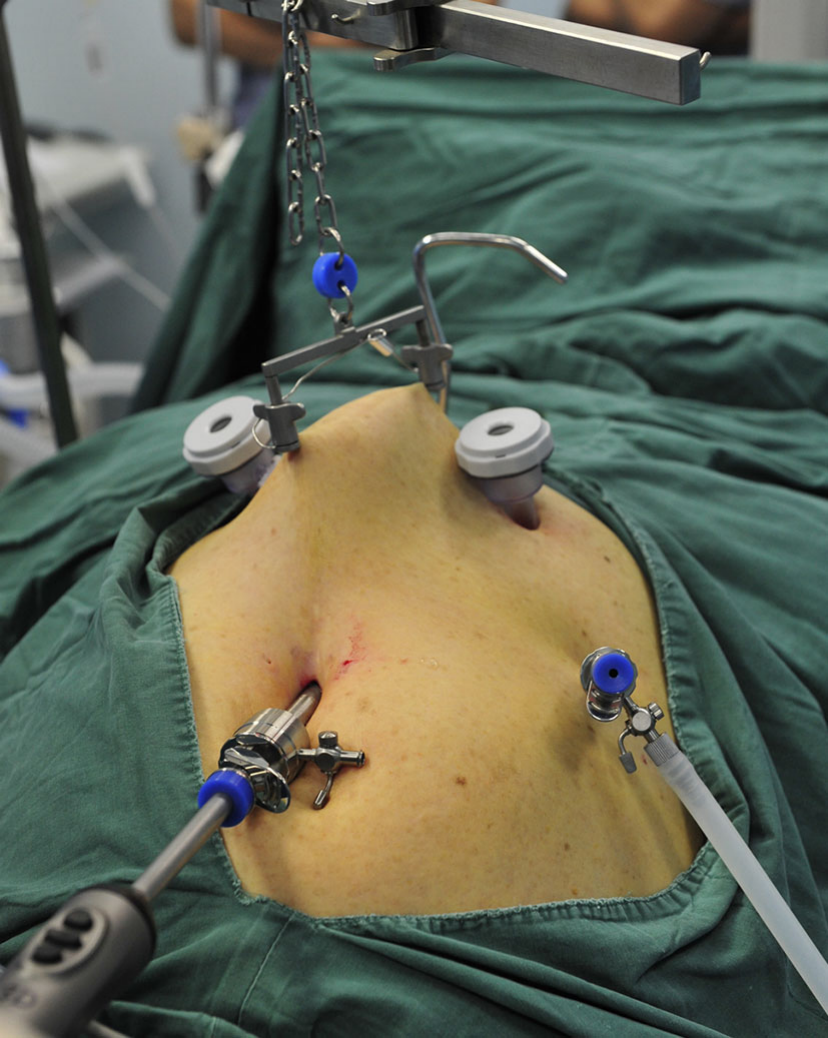
Isobaric laparoscopy using abdominal wall lifting was established

Patients were intubated under general single-lumen anesthesia, and placed in a supine position with legs spread (20–30°). At the first stage, the isobaric laparoscopy using abdominal wall lifting (Mizuho Ika Kogyo CO. LTD, Japan) was established (Fig. 2). Besides a 4-cm-long midline incision below subxyphoid made to facilitate a cotton tape and a Gauze pad into the abdomen, there were other four trocars (one 10-mm trocar and three 5-mm trocars) placed. The first step is to mobilize the stomach with the ultrasonic coagulating shears. The gastrocolic omentum is divided with care taken to preserve the right gastroepiploic arcade. Special attention should be paid when dividing the short gastric vessels. The stomach is retracted inferiorly; the short gastric vessels are identified and then divided cautiously with the ultrasonic coagulating shears. Next, the gastrohepatic ligament was opened widely. The dissection then was carried up and down the right and left crura and into the lower mediastinum. The entire periphery of the abdominal esophagus was separated.

The left gastric artery and vein could be exposed from the lesser curve view and transected at its origin with the Endo-GIA vascular stapler. A 5-cm-wide gastric tube was constructed by firing a linear stapler 3–4 times along the greater curve of the stomach from the angle at the lesser curve to the top of the fundus.

After left-sided mobilization of the cervical esophagus, the intrathoracic normal esophagus was bluntly resected from the neck to the upper mediastinum.

At the same time, the lower esophagus was transected at the gastro-esophageal junction with the ultrasonic coagulating shears. A cotton tape was sutured to the nasogastric tube and was pulled up through the mediastinal esophagus to the neck. At the transected gastro-esophageal junction, the cotton tape was attached to the distal esophagus by two stitches. A surgeon held traction to the cotton tape and pulled distal esophagus up through the posterior mediastinum to the neck, followed by a Gauze pad to press the periesophageal plane. Meanwhile, the reconstructed gastric tube was stitched to the cotton tape and 3–5 min later was drawn up through the posterior mediastinum to the neck. Esophagogastrostomy was performed in the neck, with cervical lymphadenectomy.

### Endpoint

Primary endpoints were as follows: overall survival (OS: defined as the time interval between surgery and all deaths) and postoperative laryngo-pharyngeal reflux (PLPR) (which may affect quality of life).

Secondary endpoints were as follows: the major postoperative morbidity rate (major complications occurring within 30 postoperative days and during follow-up, respectively).

### Statistical analyses

Statistical analysis was performed using the Statistical Package for the Social Sciences (SPSS, ver. 13.0). All continuous data are expressed as a mean ± standard deviation. The impact of surgery was estimated in univariate analysis. Comparisons were performed on patients with surgical treatment. Where applicable, Chi-square and Student’s *t* tests were used; survival was measured from the day of the operation until death or the last follow-up visit. Kaplan–Meier survival curves were used to compare different survival between the two groups. *P* values less than 0.05 were considered significant.

## Results

Esophagectomy by minimally invasive surgery has been successfully completed in all patients. There were no operation-related deaths and conversion to open procedure. The margin of resection was clear of tumor in all patients. Mean operative time was 131 ± 29 min in Group 1, while it was 175 ± 15 min in Group 2 (*P* = 0.005). Mean intraoperative blood loss in Group 1 was 189 ± 52 ml, significantly less than 336 ± 87 ml in Group 2(*P* = 0.001) as shown in Table 1. There was no significant difference in postoperative complications, ventilation time, ICU stay, and hospital stay between the two groups. Within 1 month after LTE, there were 7 cases having postoperative complications (Table 2): Anastomotic leakage occurred in 3 (11.1 %) cases, pulmonary complications in 2 (7.4 %) case, cardiac complications in 2 (7.4 %) cases and herniation of part of the colon into the right thorax in one case (3.7 %), 4 patients received postoperative auxiliary radiotherapy; within 1 month after CTLE, postoperative complications happened in 23 patients (Table 2): Anastomotic leakage occurred in 8 (14.3 %) cases, pulmonary complications in 8 (14.3 %), cardiac complications in 7 (12.5 %) and vocal-cord paralysis in one case (2.8 %), and nine patients received postoperative auxiliary radiotherapy. All the cases with anastomotic leakage were successfully managed within 30 days after surgery.

**Table 2:** Comparisons of postoperative complications between the two groups

	Group 1 (27 cases)	Group 2 (56 cases)	*P* Value
Complications 1 month after surgery	7	23	0.178
Pulmonary complications	2	8	
Anastomotic leakage	3	8	
Cardiac complications	2	7	
Vocal-cord paralysis	0	1	
herniation	1	0	
Wound infection	1	4	
Pulmonary complications 6 months after surgery	4	13	0.374
Anastomotic stricture	4	7	0.771

**Fig. 3:**
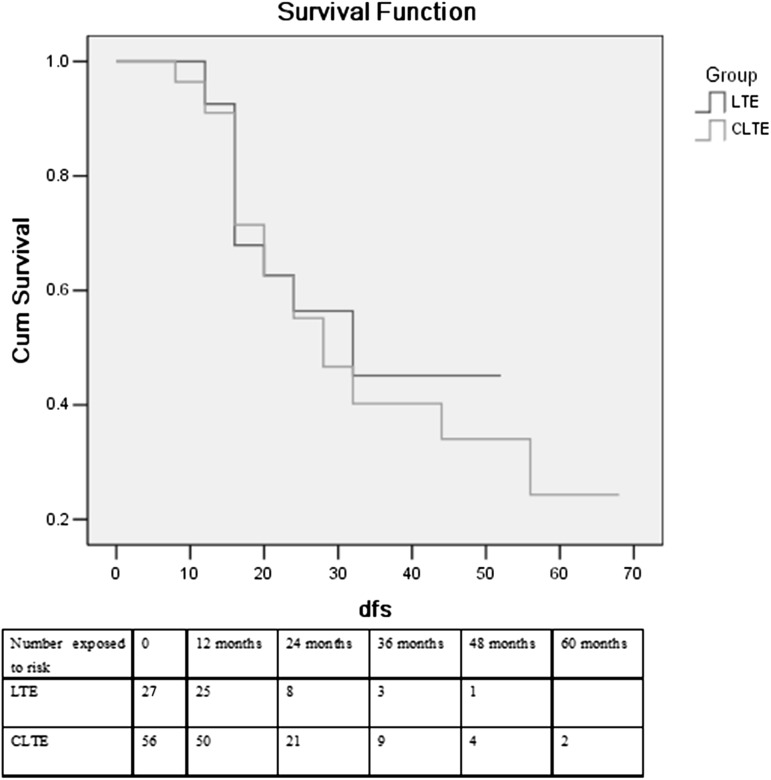
Disease-free survival curves between patients undergoing LTE and CTLE

**Fig. 4:**
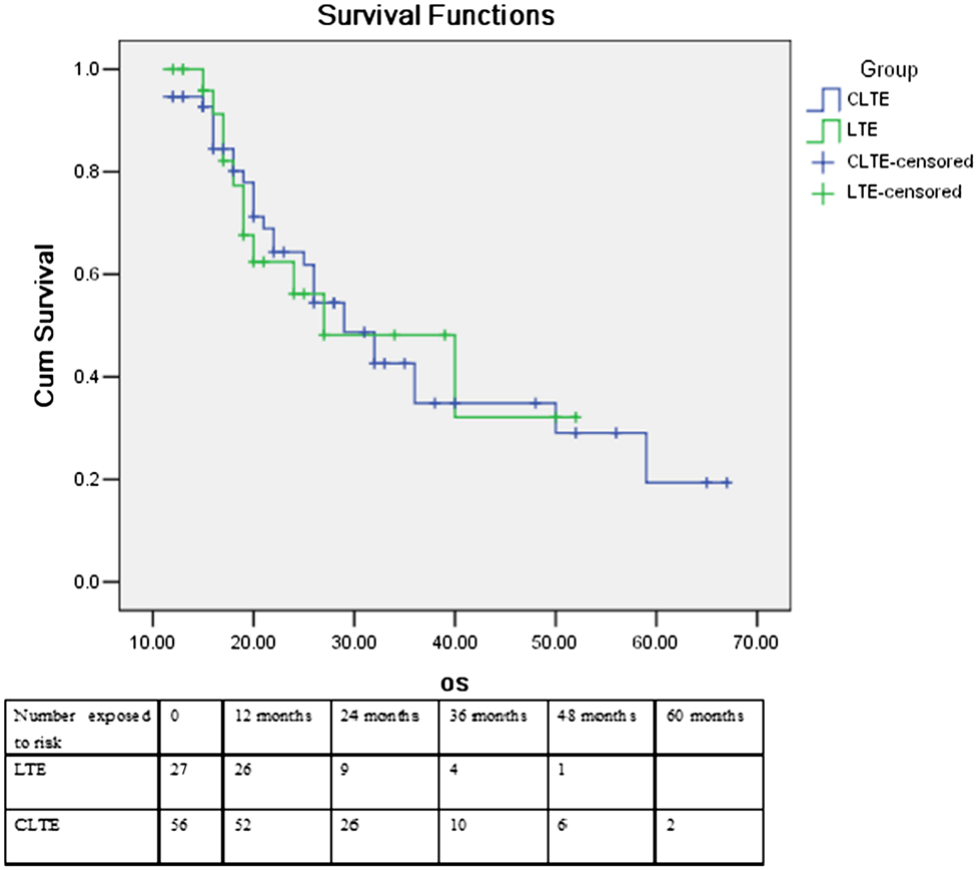
Kaplan–Meier curves showing overall survival between patients undergoing LTE and CTLE

Follow-up was complete for all patients. Patients in Group 1 were followed up for 1–4 years, while the follow-up period in Group 2 was 1–5.5 years. The median disease-free survival was 30.2 months in Group 1 and 26.4 months in Group 2 (*P* = 0.933). For Group 1 and Group 2, recurrence occurred in 48.1 % (13/27) and 51.8 % (29/56) of patients, respectively (*P* = 0.756). Survival analysis by Kaplan–Meier’s method showed no statistically significant difference between Group 1 and Group 2. The curves for disease-free (Fig. 3) and overall survival (Fig. 4) were similar after surgery without a difference favoring one technique over the other. The median overall survival was 27.2 months after the CTLE and 30.8 months after the laparoscopic transhiatal esophagectomy (*P* = 0.962). 2-, 3- and 4-year survival rates in Group 1 were 56.2, 42.5 and 32.1 %, respectively, While in Group 2, 2-, 3-, 4- and 5-year survival rates were 61.9, 34.9, 29.2 and 19.5 %, respectively. There was no significant difference in survival at 2, 3 and 4 years between the two groups. In Group 1, among 11 deaths, eight were associated with recurrence or metastasis and three were due to heart failure. In Group 2, 23 patients died of recurrence or metastasis, three deaths were attributable to pulmonary complications, two died of portal vein thrombosis, and one death was related to heart failure.

All the patients complained about heartburn and regurgitation from time to time after surgery. Major complications more than 6 months after surgery were pneumonia and anastomotic stricture (Table 2): in Group 1, there were 4 patients having pulmonary complications at least one time and 4 patients experiencing anastomotic stricture, while in Group 2, 13 patients had postoperative pulmonary complications (*P* = 0.374) and seven had postoperative anastomotic stricture (*P* = 0.771). Anastomotic stricture was managed successful by anastomotic dilatations.

**Fig. 5:**
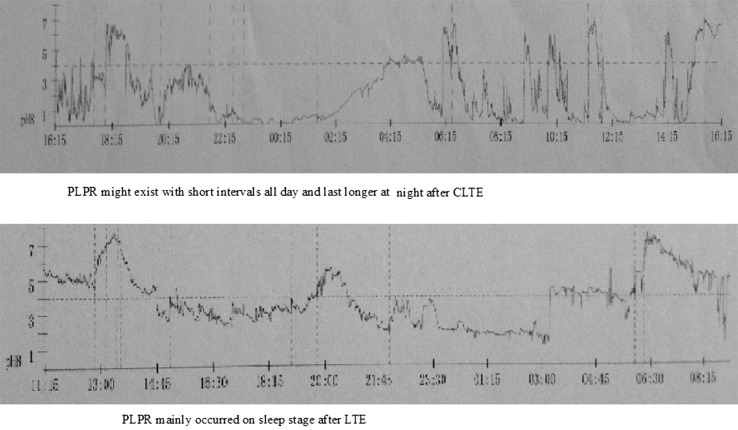
Comparison of postoperative 24-h pH monitoring 6 months after CTLE or LTE

Results of postoperative 24-h pH monitoring and manometry were documented as shown in Table 3. Maybe due to short length of residual esophagus, no consistent motility pattern was detected. PLPR was more severe in Group 2 than in Group 1: By univariate analysis, the total number of reflux events (pH < 4), the number of reflux episodes (lasting > 5 min) and the reflux time in Group 1 were less than those in Group 2 (*P* = 0.00), while the longest episode of reflux in Group 1 was longer than that in Group 2 (*P* = 0.01). 24-hour ambulatory esophageal pH monitoring also revealed that for patients in Group 1, PLPR mainly occurred at sleep stage, while for patients in Group 2, PLPR might exist with short intervals all day and last longer at night (Fig. 5).

**Table 3:** Multiple comparisons of 24-h pH monitoring and manometry (more than 6 months after surgery) between Group 1 and Group 2: $(\bar{x} \pm s)$

	Group 1 (21 cases)	Group 2 (45 cases)	*P* value
Total number of reflux events (pH < 4)	15.33 ± 2.82	28.76 ± 4.57	0.001
Number of reflux episodes (lasting > 5 min)	5.19 ± 1.57	15.07 ± 1.85	0.000
The reflux time	2.28 ± 0.59	5.02 ± 0.50	0.000
The longest episode of reflux (min)	29.50 ± 4.83	22.51 ± 3.09	0.001
UESP (mm Hg)	12.34 ± 1.35	11.97 ± 1.00	0.217
UESL (cm)	1.88 ± 0.46	1.75 ± 0.34	0.227

## Discussion

Up to now, esophagectomy with curative intent has been regarded as the most effective treatment for early-stage esophageal carcinoma [5–7]. Conventional approaches, involving laparotomy and/or thoracotomy, are associated with high incidence of morbidities, which delay the recovery. Especially for upper esophageal carcinoma, cervicothoracoabdominal esophagectomy is one of the most complex surgical procedures with great trauma, making some surgeons and patients dismay.

A great improvement in esophagectomy is the application of minimally invasive techniques. Since Swanstrom and Hansen introduced their experience on a completely laparoscopic approach to esophageal cancer in 1997 [13], the application of minimally invasive surgery for esophageal cancer has become rapidly widespread in recent years.

CTLE has been performed in our department in 2009, and it showed significant advantages over open procedure in mortality and morbidity as other studies reported [14]. Due to the use of advanced instruments, current minimally invasive esophagectomies hold normal laparoscopic or thoracoscopic advantages. Both CTLE and LTE were associated with short period of ICU and hospital stay, relatively quick recovery, small incisions, and so on.

But in recent years, the aging demographics and the growing number of the elderly patients with upper esophageal carcinoma were becoming surgical problems we faced. Could we develop other minimally invasive techniques for upper esophageal carcinoma to decrease early postoperative risk? Can we develop some kind of more minimally invasive esophagectomy which the elderly or patients with severe preoperative pulmonary dysfunction could withstand? Would neoadjuvant chemotherapy be helpful to improve long-term outcome of minimally invasive esophagectomy without formal lymphadenectomy? Based on the questions mentioned above, the gasless laparoscopic transhiatal esophagectomy was developed.

**Fig. 6:**
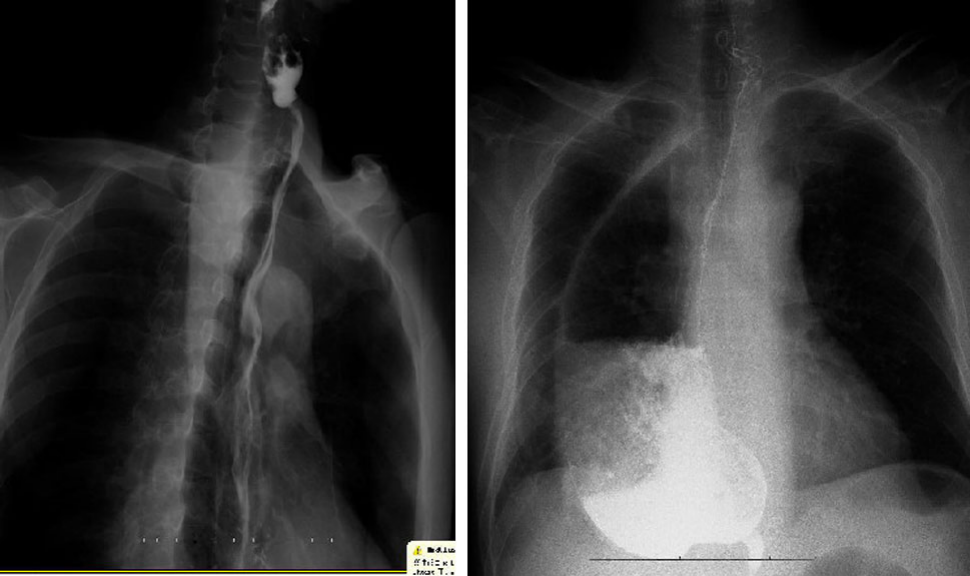
Barium esophagram showed that the constructed gastric tube was limited in the posterior mediastinum 6 months after LTE (*left*), while it might expand in the right plural cavity 6 months after CTLE (*right*)

Compared with CTLE, LTE had showed its own advantages. First of all, the adverse effects of CO2 insufflation and single-lung ventilation are eliminated. As life expectancy rises in China, many patients 80 years old and higher with esophageal cancer expect to prolong their life by surgical treatment. These patients do not have adequate pulmonary reserve [15], so it seems difficult for them to tolerate pneumoperitoneum and single-lung ventilation. The procedure of LTE has little to do with the thoracic cavity and lung. So poor pulmonary function was no obstacle to surgical treatment to upper esophageal carcinoma any more. In Group 1, eight patients with low pulmonary function (80 years old or greater) successfully underwent LTE. LTE can be safely performed for upper esophageal carcinoma with severe preoperative pulmonary dysfunction. Secondly, the peritoneal cavity does not need to be sealed airtight when performing LTE. This facilitates several steps of the procedure. The operating time is decreased because an optimal view can be maintained even during irrigation suction. In our series, mean operative time in Group 1 was only 131 ± 29 min, significantly lower than that in Group 2 (*P* = 0.000). What is more, benefits of LTE were also linked with a lower mean intraoperative blood loss. Mean intraoperative blood loss of LTE was only 189 ± 52 ml, even significantly lower than CTLE (*P* = 0.001). Finally, severe PLPR happened less in Group 1 than that in Group 2. PLPR occurred every day in patients undergoing CTLE and LTE. PLPR had a major effect on quality of life of patients experiencing esophagectomy. High incidence of postoperative pulmonary complications was mainly related to severe PLPR. Tracking PLPR can provide important clues as to postoperative pulmonary complication and factors influencing postoperative quality of life [16]. It should be noted that in our series, incidence rates of postoperative pulmonary complications, including those occurring within 30 postoperative days and during follow-up, are relatively high. In Group 2, 21 (37.5 %) patients had postoperative pulmonary complications, while in Group 1, there were six (22.2 %) patients having pulmonary complications at least one time (*P* = 0.164). All the patients experiencing CTLE or LTE complained about heartburn and regurgitation from time to time. In Group 2, it was more severe than in Group 1. In Group 1, PLPR mainly occurred at sleep stage, while for patients in Group 2, PLPR might exist all the day only with short intervals and last longer at night. This might lead to higher incidence of postoperative pulmonary complications in Group 2. Mediastinal tissue press may contribute to reducing occurrence of PLPR and postoperative pulmonary complications in patients undergoing LTE (Fig. 6).

For a long period of time, the transhiatal esophagectomy has been regarded as a controversial procedure because of failure to do extensive lymphadenectomy. In order to try to decrease the possible influence of inadequate lymph-node dissection, patients undergoing LTE in this study received neoadjuvant and adjuvant chemotherapy. It is uncertain whether neoadjuvant chemotherapy followed by esophagectomy might lead to greater long-term survival [17–19]. But survival analysis by Kaplan–Meier’s method demonstrated that overall survival and disease-free survival in Group 1 seem to be similar to those in Group 2. The median overall survival of Group 1 and Group 2 reached up to 30.8 and 27.2 months, respectively (*P* = 0.962). Furthermore, neoadjuvant chemotherapy effectively downstaging esophageal carcinoma with local advance was observed in 11 cases. Downstaging these tumors made them more resectable.

In conclusion, compared with CTLE, LTE is a more minimally invasive approach to effectively treat patients with upper esophageal carcinoma. Laryngo-pharyngeal reflux after LTE was less severe than that after CTLE, which might lower incidence of pulmonary complications. For the elderly patients, LTE seems more suitable.
